# A Randomized Factorial Trial of Varenicline, Nicotine Lozenges, and Smartphone Medication Adherence Reminders for Smoking Cessation: Protocol for the COMBO II Study

**DOI:** 10.2196/108379

**Published:** 2026-11-02

**Authors:** Nathalia M Machado, Jasjit S Ahluwalia, Michael S Businelle, Summer G Frank-Pearce, Irina Baranskaya, Emily T Hébert, Munjireen S Sifat, Shannon H Gwin, Roma Thakur, Sharon Glassman, Henry W Kinnard, Jr, Gaurav Kumar, Deeksha Gupta, Darla E Kendzor

**Affiliations:** 1 TSET Health Promotion Research Center, Stephenson Cancer Center, University of Oklahoma Health Campus, 655 Research Parkway, Oklahoma City, OK, 73104, United States, 1 (405) 271-6872; 2 Department of Internal Medicine, University of Oklahoma Health Campus, Tulsa, OK, United States; 3 Department of Behavioral and Social Sciences and Center for Alcohol and Addiction Studies, Brown University School of Public Health, Providence, RI, United States; 4 Department of Medicine, Alpert Medical School, Brown University, Providence, RI, United States; 5 Department of Family and Preventive Medicine, University of Oklahoma Health Campus, Oklahoma City, OK, United States; 6 Department of Biostatistics and Epidemiology, Hudson College of Public Health, University of Oklahoma Health Campus, Oklahoma City, OK, United States; 7 Department of Psychiatry and Behavioral Sciences, University of Oklahoma Health Campus, Oklahoma City, OK, United States; 8 College of Pharmacy, University of Oklahoma Health Campus, Oklahoma City, OK, United States

**Keywords:** smoking cessation, varenicline, nicotine replacement therapy, smartphone, mobile health, randomized controlled trial, medication adherence

## Abstract

**Background:**

Smoking remains the leading preventable cause of cancer mortality in the United States. Varenicline is the most effective monotherapy for smoking cessation, and combining it with nicotine replacement therapy (NRT) may further improve cessation rates. Smartphone-based medication adherence interventions could enhance pharmacotherapy adherence and cessation outcomes.

**Objective:**

This trial will evaluate the efficacy of varenicline plus nicotine lozenges and a smartphone adherence intervention for promoting smoking cessation.

**Methods:**

This fully remote, 2 × 2 factorial randomized controlled trial will enroll 496 adults from Oklahoma who smoke cigarettes daily. Participants will be randomized to 1 of 4 conditions: (1) varenicline (VAR) + nicotine lozenges (NRT) versus VAR alone and (2) a smartphone-based smoking cessation app with adherence components (COMBO-A [adherence]) or without adherence components (COMBO-B [basic]). The primary outcomes are biochemically verified 7-day point prevalence abstinence at 26 weeks post quit date and medication adherence during the treatment period. Secondary outcomes include withdrawal symptoms and medication side effects over the 12-week treatment period.

**Results:**

Study recruitment began in July 2026. As of the date of this paper’s submission, 8 participants have been enrolled. Enrollment is expected to be completed by December 2028, with primary data collection and follow-up completed by June 2029. Primary study results are anticipated later in 2029.

**Conclusions:**

If effective, VAR+NRT would offer a new evidence-based cessation treatment option. If the smartphone adherence intervention improves medication adherence and cessation, it could be adapted for other pharmacotherapies and widely implemented. Ultimately, this research aims to enhance cessation efforts and reduce cancer risk among adults who smoke.

## Introduction

Although smoking prevalence among US adults has declined from 42.4% in 1965 to 11.6% in 2022 [1], smoking remains the leading preventable cause of cancer and mortality in the United States and worldwide [2]. Quitting smoking is essential to reducing tobacco-related morbidity and mortality and is beneficial to one’s health at any age, with potential gains of up to approximately 10 years of life expectancy [3-5]. There are several effective and US Food and Drug Administration–approved pharmacotherapies to assist individuals in quitting smoking. Varenicline is the most effective single medication for smoking cessation [6]. The combination of pharmacotherapy with behavioral support further improves cessation outcomes [7,8]. While clinical trials [9,10] and meta-analyses [11,12] have demonstrated that combining varenicline with nicotine patches improves quit rates, no full-scale randomized trial has evaluated the combined effectiveness of varenicline and oral nicotine replacement therapy (NRT) for smoking cessation. Oral NRT, such as nicotine lozenges [13], offers a rapid and controlled method to deliver nicotine, providing acute relief from cravings and withdrawal symptoms as they arise, in contrast with the continuous, passive, and slow-acting delivery of the nicotine patch [12]. The pilot study that led to the current trial [14] descriptively found higher biochemically verified abstinence rates among participants who received varenicline (VAR) plus oral NRT (nicotine lozenges or gum) compared with VAR alone at all follow-up points through 26 weeks after the scheduled quit day.

Nevertheless, adherence to tobacco cessation medications remains a significant challenge. Greater adherence, particularly in the early weeks of a quit attempt, is associated with higher cessation rates [15]. However, most individuals using oral NRT do not follow recommended dosing guidelines and often underdose NRT and discontinue use prematurely, reducing the likelihood of successful cessation [16,17]. Common barriers to medication adherence include forgetfulness, the belief that smoking cessation medications are not needed, concerns about side effects, low confidence in medication effectiveness, perceived lack of willpower, and external factors such as cost or limited pharmacy access [18,19]. Improving adherence improves cessation outcomes [15] and strengthens the ability to evaluate the efficacy of combination pharmacotherapies by reducing the confounding effects of nonadherence [20,21].

Smartphone-based and other digital interventions can reach individuals across various settings and thus have the potential to enhance day-to-day pharmacotherapy adherence [22-24]. In 2024, approximately 91% of US adults owned a smartphone [25], highlighting the promise of smartphone interventions as a means to increase treatment reach and engagement. Findings from a review and meta-analysis [26] suggested that smartphone-based interventions can be effective for promoting smoking cessation, especially when integrated with pharmacotherapy-based approaches. Likewise, preliminary findings from our pilot study indicated that participants who received smartphone-based medication reminders had descriptively higher smoking abstinence rates, greater adherence to varenicline, and more consistent use of oral NRT compared with those who did not receive reminders [14].

This randomized controlled trial will evaluate the efficacy of combining varenicline, nicotine lozenges, and a smartphone-based intervention to promote medication adherence and smoking cessation among 496 adults who smoke daily. The trial will use a 2 × 2 factorial design in which participants will be randomized to 4 different combinations of a pharmacological treatment (VAR or VAR + nicotine lozenges [NRT]) and a smartphone medication adherence intervention (COMBO-A [smoking cessation app with adherence components] vs COMBO-B [basic smoking cessation app]). It is hypothesized that (1) smoking cessation rates will be significantly greater among participants assigned to VAR + NRT than among those assigned to VAR and (2) medication adherence and smoking cessation rates will be greater among participants assigned to COMBO-A than among those randomized to COMBO-B. Potential interactions between medication type (VAR + NRT vs VAR) and smartphone intervention (COMBO-A vs COMBO-B) on smoking cessation and medication adherence will be explored. Finally, exploratory analyses will compare the influences of medication type and smartphone intervention on weekly symptoms (withdrawal symptoms and medication side effects) through the end of treatment (12 wk after the quit date). The overarching goal is to improve smoking cessation treatment and reduce cancer risk.

## Methods

### Design

This randomized clinical trial will employ a 2 × 2 factorial design to evaluate the efficacy of 2 treatment factors on smoking cessation and medication adherence. Participants will be randomized to 1 of 4 combinations of 2 treatment factors: (1) pharmacological treatment (VAR vs VAR+nicotine lozenges [VAR+NRT]) and (2) smartphone-based smoking cessation app (with adherence components [COMBO-A] vs without adherence components [COMBO-B]). All participants, regardless of treatment group assignment, will be offered tobacco cessation counseling sessions via telephone with a tobacco treatment specialist. This factorial design will allow for an efficient evaluation of both treatment factors simultaneously rather than conducting separate studies, optimizing statistical power and resource use. This design will also allow for the exploratory analysis of the potential interaction between treatment factors. Table 1 illustrates the study design and treatment groups. The study protocol was prepared in accordance with the SPIRIT guidelines (Checklist 1).

**Table 1.** Factorial design (2 × 2) and intervention groups.

	Smartphone-based smoking cessation intervention	
Medication type	COMBO-A^a^ (adherence)	COMBO-B^b^ (basic)
VAR^c^	VAR+COMBO A (n=124)	VAR+COMBO B (n=124)
VAR+NRT^d^	VAR+NRT+COMBO-A (n=124)	VAR+NRT+COMBO-B (n=124)

a COMBO-A: smartphone intervention with medication adherence components.

b COMBO-B: smartphone intervention without medication adherence components.

c VAR: varenicline.

d NRT: nicotine replacement therapy (lozenge).

### Participants

Participants (N=496) will be recruited statewide in Oklahoma through social media advertisements, electronic medical record referrals for tobacco treatment at the University of Oklahoma Health Campus, and print advertising. Eligibility criteria include (1) documentation of identity (to prevent fraudulent enrollment) and Oklahoma residency (required for prescription regulations), (2) age 18 years or older, (3) smoking at least 5 cigarettes per day, (4) breath carbon monoxide (CO) sample ≥6 ppm to confirm current smoking, (5) willingness to schedule a smoking cessation attempt within the next 1 to 2 weeks, (6) willingness to abstain from smoking cannabis or other combustible products during the study to prevent CO elevation that could interfere with smoking abstinence verification, (7) willingness and eligibility to use varenicline and nicotine lozenges, (8) willingness to participate in the study for approximately 6 months, and (9) English literacy above the sixth-grade level (assessed via the Rapid Estimate of Adult Literacy in Medicine-Short Form [REALM-SF]) [27] to verify the ability to read the intervention materials and complete assessments. Participants will be excluded if they (1) have any varenicline contraindication (eg, history of seizures or history of allergic reaction to varenicline), (2) are pregnant or planning to become pregnant, or are currently breastfeeding, (3) are unwilling or unable to use varenicline or nicotine lozenges for other reasons (eg, severe dry mouth or a past history of severe medication side effects), (4) have other medical contraindications at the discretion of the prescribing physician (eg, uncontrolled hypertension or recent myocardial infarction), (5) have ≥weekly use of combustible cannabis during the past 30 days and score ≥2 on the Cannabis Use Disorder Identification Test-Short Form (CUDIT-SF) [28], indicating potential difficulty in abstaining from smoking cannabis during the study, (6) do not own a smartphone that is compatible with the study app, (7) have used an US Food and Drug Administration–approved smoking cessation medication within the past 30 days, or (8) have used oral nicotine products (eg, nicotine pouches, chewing tobacco, or snus) more than once per week (≥5 times) during the past 30 days. This study focuses on smoking cessation specifically, and thus, individuals who use other noncombustible tobacco products will not be excluded from participation.

### Procedures

Participant eligibility will be assessed first via a REDCap [29,30] screening form accessed through social media advertisements or during a phone screening call. Those meeting preliminary criteria will be scheduled for a telephone appointment with a study staff member (Baseline Part 1). During this appointment, participants will complete the informed consent and Health Insurance Portability and Accountability Act authorization forms electronically, provide a photo of a valid government-issued identification to verify identity and Oklahoma residency, and complete screening measures, including tobacco use history and the REALM-SF [31]. Participants will be assisted with downloading and initializing the study smartphone app. Participants will be instructed to complete the full baseline survey via the smartphone app within 24 hours (with up to 7 days allowed before being considered ineligible). Following completion of the baseline survey, study materials, including a portable breath CO monitor and a reloadable study credit card, will be mailed to participants. For those who do not own a compatible phone, a smartphone containing the study app will be provided.

Following receipt of study materials, participants will complete a videoconferencing appointment with study staff (Baseline Part 2A), during which they will submit a CO breath sample to verify current smoking status and confirm identity. This step serves as a secondary identity verification to ensure that the individual completing the breath sample matches the participant verified during the initial enrollment appointment. Participants will then complete a separate videoconferencing appointment with a study physician to determine eligibility for varenicline use (Baseline Part 2B). If medically eligible, varenicline will be prescribed and dispensed through a collaborating campus pharmacy and mailed to participants. Participants randomized to the VAR+NRT condition will also be mailed nicotine lozenges. Participants who do not meet eligibility criteria at any point during baseline procedures will be excluded and will not proceed to randomization.

A counseling and randomization appointment (prequit appointment) will be scheduled approximately 1 week after completion of baseline procedures. Participants will be randomized to a treatment condition using a stratified block randomization approach (block size=8) to ensure balance between study arms over time. Randomization will be stratified by sex (male vs female), race/ethnicity (White vs minoritized race/ethnicity), and number of cigarettes smoked per day (<10 vs ≥10). During this appointment, participants will complete their first telephone counseling session with a tobacco treatment specialist and will be informed of their randomized treatment assignment. Participants will begin taking varenicline and completing daily smartphone assessments the day following this visit. A 7-day prequit medication titration period will occur during this time, during which daily assessments will also be collected. The scheduled quit date will occur on day 8 (ie, the day following the seventh day of medication titration). Participants assigned to VAR+NRT will be instructed to begin nicotine lozenges on the quit day.

Participants will complete 1 prequit counseling session followed by 5 weekly postquit sessions (6 total counseling sessions). Varenicline treatment will continue for 13 weeks (including a 1-week prequit titration and 12 weeks postquit), and nicotine lozenges will be used for 12 weeks postquit for participants assigned to combination therapy. Daily smartphone-based assessments will be completed beginning 1 week prior to the scheduled quit date through 12 weeks postquit (13 weeks total). Smartphone-based follow-up assessments are scheduled for weeks 1‐12, 17, 20, and 23 after the scheduled quit day, with a final follow-up assessment at 26 weeks after the quit day. The interventions and assessments are described in detail below and presented in Figure 1.

**Figure 1.**
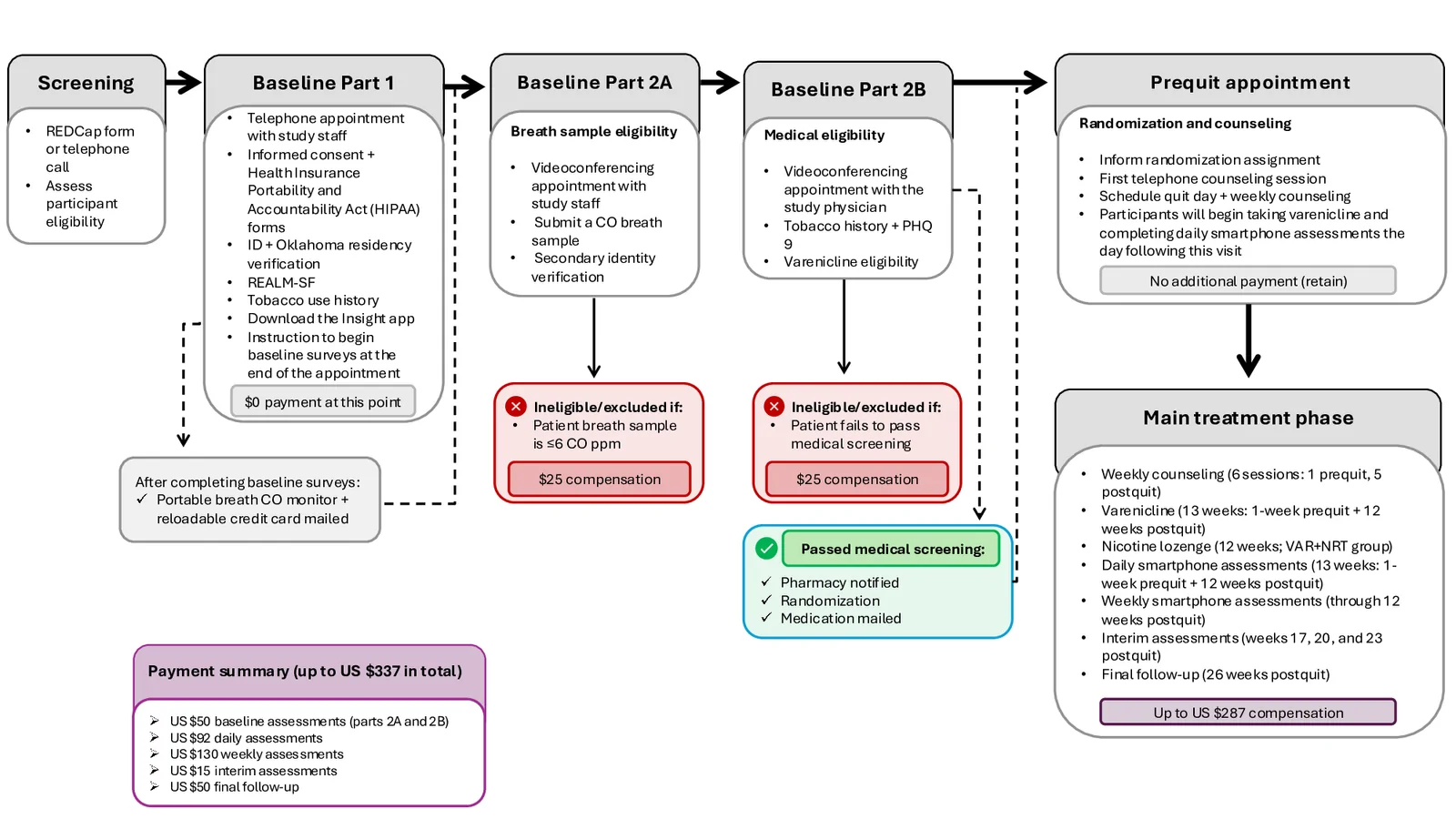
Study procedures and compensation structure. CO: carbon monoxide; NRT: nicotine replacement therapy (lozenge); PHQ: Patient Health Questionnaire; REALM-SF: Rapid Estimate of Adult Literacy in Medicine-Short Form.

### Interventions

#### General Intervention Components

This study will offer both counseling and pharmacotherapy, consistent with current recommendations for optimal tobacco treatment [16]. All participants will receive 13 weeks of varenicline, beginning with the standard 1-week prequit titration (days 1‐3: 0.5 mg once daily; days 4‐7: 0.5 mg twice daily), followed by 1 mg twice daily for an additional 12 weeks. Additionally, all participants will have access to the study smartphone intervention app (with or without medication adherence components). Finally, all participants will receive 6 weekly telephone counseling sessions with a tobacco treatment specialist from the Tobacco Treatment Research Program [32] at the University of Oklahoma Health Campus. The initial counseling session will occur approximately 1 week prior to the scheduled quit date and will continue weekly through 4 weeks postquit (6 sessions total lasting 15‐30 min each). Consistent with clinical practice guidelines [7], counseling will focus on education, skill-building, and support, employing cognitive-behavioral and motivational strategies.

#### Pharmacological Treatment (VAR+NRT or VAR)

All participants will receive a 13-week supply of varenicline, which will be mailed prior to the scheduled quit date. Daily doses will be titrated during the week prior to the scheduled quit day (days 1‐7), as described above, and will continue at the full dose for 12 additional weeks (days 8‐91). Participants randomized to the combination therapy group will also be mailed a 12-week supply of nicotine lozenges (NRT). Participants will receive 5 boxes of nicotine lozenges (108 pieces per box; 540 pieces total) in a single mailing. This quantity provides a supply that supports flexible ad libitum dosing while also supporting optimal dosing based on package recommendations [33]. Participants may request additional lozenges during the first 12 weeks after the scheduled quit date if needed, and additional supply requests will be documented. Participants will be advised to begin using the lozenges on the quit day and continue through 12 weeks postquit (days 8‐91), following the standard nicotine lozenge dosing instructions printed on the package.

#### Smartphone-Based Smoking Cessation Intervention (COMBO-A or COMBO-B)

All participants will download the smartphone app, which will be configured via the Insight Mobile Health Platform [34]. For all participants, the app will provide access to core study features throughout participation, including study information, counseling appointment schedules, medication instructions, treatment topics, compensation tracking, troubleshooting resources, and the ability to contact study staff. Participants will also complete study assessments (eg, baseline, daily, weekly, interim, and follow-up surveys) and CO breath testing through the app at scheduled time points. For participants assigned to the COMBO-A intervention, additional medication adherence components will be activated, including automated medication reminders and graphical feedback on medication adherence. These features are designed to support consistent use of varenicline and nicotine lozenges. See Table 2 for a detailed description of intervention components by study group.

**Table 2.** Treatment components by treatment group assignment.

Treatment component	VAR^a^+COMBO-B^b^	VAR+COMBO-A^c^	VAR+NRT^d^+COMBO-B	VAR+NRT+COMBO-A
Smartphone app	✓	✓	✓	✓
Study information	✓	✓	✓	✓
Telephone counseling schedule	✓	✓	✓	✓
Medication instructions	✓	✓	✓	✓
Smoking cessation treatment topics	✓	✓	✓	✓
Varenicline reminders (twice daily)	—^e^	✓	—	✓
Nicotine lozenge reminders	—	—	—	✓
Graphical medication adherence tracking	—	✓	—	✓
Study compensation tracking	✓	✓	✓	✓
Troubleshooting topics	✓	✓	✓	✓
Study contact information	✓	✓	✓	✓
Other treatment components				
Telephone counseling	✓	✓	✓	✓
Varenicline	✓	✓	✓	✓
Nicotine lozenges	—	—	✓	✓

a VAR: varenicline.

b COMBO-B: smartphone intervention without medication adherence components.

c COMBO-A: smartphone intervention with medication adherence components.

d NRT: nicotine replacement therapy (lozenge).

e Not applicable.

All participants will be required to specify a 13-hour wake period. During this period, participants assigned to the COMBO-A intervention will receive smartphone medication reminders aligned with the varenicline dosing schedule. Reminders will appear 30 minutes after participants’ self-reported wake time (AM reminder) and 2 hours before bedtime (PM reminder). Along with the reminder, participants will be asked whether they have taken their pill. Participants who indicate that they are going to take their pill or have already taken their pill will receive an affirmation message. If the participant has not yet taken the dose, follow-up prompts will appear at 30-minute intervals, for up to 3 follow-up prompts. Following the final prompt, participants who indicate that they have not taken their pill will be asked about the main reason for the missed dose and will receive an adherence-promotion message tailored to their response. See Table 3 for example messaging and assessments.

**Table 3.** Examples of varenicline medication reminders and follow-up questions/messages.

Reminder type	Message	Response option
Varenicline		
AM varenicline instructions (days 1‐3 [titration week])	You should take ONE 0.5 mg varenicline pill per day for the first 3 days.	N/A^a^
AM varenicline reminder (days 1‐3 [titration week])	Don’t forget to take your varenicline (pill) this morning! Instructions: Take one 0.5 mg pill in the morning after eating and with a full glass of water. Will you take your morning varenicline pill now?	1=Yes, I will take my morning pill now; 2=Yes, I already took my morning pill; 3=No, I don’t plan to take my morning pill
Affirmation message	Nice job! Taking your varenicline (pill) as prescribed increases your chances of quitting smoking!	N/A
Assessment of reason for not taking varenicline pill	What is the main reason why you don’t plan to take your varenicline (pill) now?	1=I do not have my pills with me; 2=Unpleasant side effects; 3=I prefer to quit without medication*; 4=I do not think the pills are helping me; 5=I am currently smoking; 6=Other
Follow-up message	[*If the participant selects reason 3 for not taking their medication] Studies show that the chances of quitting are up to 5 times higher with medication than without. Quitting is difficult—medication can make the process a little bit easier!	N/A
Nicotine lozenges		
Morning lozenge reminder (days 8‐49 [first 6 weeks after quit day])	For the best chances of success, use a nicotine lozenge every 1‐2 hours during the first 6 weeks of quitting. Try to use at least 9 lozenges (but no more than 20) per day! When did you last use a lozenge?	1=1‐2 hours ago; 2=3‐4 hours ago; 3=5‐6 hours ago; 4=7‐8 hours ago; 5=More than 8 hours ago; 6=I have not used a lozenge today
Assessment of reason for not using nicotine lozenge	What is the main reason that you have not used a lozenge in the past 2 hours?	1=It is morning, and I haven’t gotten to it yet; 2=I forgot**; 3=I did not have my lozenges with me; 4=Unpleasant side effects; 5=I prefer to quit without medication; 6=I do not think the lozenges are helping me; 7=I do not like the taste of the lozenges; 8=I am currently smoking; 9=Other
Follow-up message	[**If the participant selects reason 2 for not using a lozenge] No problem! Use a lozenge now and try to use one every 1‐2 hours. Plan to use a lozenge when you receive a phone reminder and whenever you feel a craving.	N/A

a N/A: not applicable.

Participants randomized to VAR+NRT+COMBO-A will receive additional smartphone-based medication reminders corresponding to the standard nicotine lozenge dosing schedule, in addition to varenicline reminders. Beginning on the quit day, participants will receive reminders to use nicotine lozenges every 1 to 2 hours (6 reminders per day), consistent with recommended dosing guidelines [35], for 6 weeks (days 8‐49). During postquit weeks 7 to 9 (days 50‐70), participants will receive reminders to use nicotine lozenges every 2 to 4 hours (4 reminders per day). Finally, during postquit weeks 10 to 12 (days 71‐91), participants will receive reminders to use nicotine lozenges every 4 to 8 hours (3 reminders per day). After each reminder, participants will be asked when they last used a nicotine lozenge. Participants who report that they have not used a lozenge in more than 8 hours will be asked about the main reason for not using a lozenge and will receive an adherence-promotion message tailored to their response. See Table 3 for example messaging and assessments.

### Blinding

Participant blinding is not feasible because participants will be aware of whether or not they are provided with nicotine lozenges (in addition to the varenicline provided to all participants), and they will know whether their smartphone app has medication adherence components. Counselors, outcome assessors, and statisticians will also not be blinded to treatment assignment. Potential bias associated with the open-label design will be minimized through standardized intervention and assessment procedures and the use of biochemically verified smoking abstinence as the primary outcome.

### Measures

#### Assessment Schedule and Participant Compensation

Participants will be prompted to complete an initial baseline assessment, daily and weekly assessments each morning through 12 weeks postquit, interim follow-up assessments at 17, 20, and 23 weeks postquit, and a final follow-up at 26 weeks postquit. All surveys will be completed via smartphone. Participants will be compensated up to US $337 in total via a reloadable study credit card. Compensation includes US $1 per day for completing daily diary assessments during the first 13 weeks (up to US $92), US $50 for completing the baseline 2A and 2B assessments (US $25 each), US $10 per week for completing weekly questionnaires with CO breath sample submissions during the first 13 weeks (up to US $130), US $5 for completing each interim assessment (up to US $15), and US $50 for completing the final follow-up assessment at 26 weeks postquit. A summary of all measures and the assessment schedule is presented in Table 4. Assessments are described in detail below.

**Table 4.** Study assessment timeline.

Study assessments	Baseline and/or screening (prequit)	Daily	Weekly (1‐13 wk)	Interim assessment (17, 20, 23 wk postquit)	Final follow-up (26 wk postquit)
Sociodemographic and background information					
Sociodemographic/background characteristics	✓				
Rapid Estimate of Adult Literacy in Medicine-Short Form [31]	✓				
Financial Chronic Strain Scale [36]	✓				✓
Food Security Survey [37]	✓				✓
Politics and religion [38,39]	✓				
Tobacco use					
Tobacco use history	✓				
Heaviness of Smoking Index [40]	✓				
Brief Wisconsin Smoking Withdrawal Scale (WSWS2-B) [41]	✓	✓			
Smoking/tobacco use status	✓	✓	✓	✓	✓
Carbon monoxide breath sample	✓		✓	✓	✓
Physical/mental health and substance use					
Medical screening	✓				
Patient Health Questionnaire (PHQ-8 or PHQ-9)^a^ [42,43]	✓		✓^b^	✓	✓
Center for Epidemiological Studies-Depression-10 Scale [44]	✓		✓^c^	✓	✓
Alcohol Use Disorders Identification Test-Consumption [45]	✓				✓
Alcohol quantity and frequency (previous week) [46]	✓		✓^d^	✓	✓
Alcohol use (previous day)		✓			
Marijuana/cannabis use behavior	✓				
Cannabis use (previous day or previous week)		✓	✓^d^	✓	✓
Cannabis Use Disorder Identification Test-Short Form [28]	✓				✓
Self-rated health [47]	✓		✓^c^		✓
Height	✓				
Weight	✓		✓		✓
Treatment moderators/mechanisms					
Motivation to quit slider	✓				
Motivation/importance/readiness [48]	✓		✓	✓	✓
Smoking self-efficacy/temptation short form [49]	✓		✓^b^		
Medication use/reasons for not using		✓			
Adherence to Refills and Medications Scale (adapted [50,51])			✓		
Symptoms and medication side effects			✓		
Other measures					
Sleep duration and quality (adapted [52])	✓	✓			
Pain (adapted [53,54])		✓			
Political stress			✓		
Treatment fidelity/usability					
Credibility/Expectancy Questionnaire [55]			✓^e^		
Working Alliance Inventory-Short Revised [56,57]			✓^f^		
System Usability Scale [58,59]			✓^g^		

a The PHQ-9 is administered just prior to the psychiatrist consultation (baseline), and the PHQ-8 (with the suicidal ideation item omitted) is administered thereafter.

b Assessed at 2, 4, 8, and 12 weeks postquit.

c Assessed at 2, 4, 6, 8, 10, and 12 weeks postquit.

d Assessed at 4, 8, and 12 weeks postquit.

e Assessed from the quit day through 4 weeks postquit.

f Assessed at 1 and 4 weeks postquit.

g Assessed at 12 weeks postquit.

#### Screening

Screening assessments will be conducted prior to baseline to determine study eligibility. Initial screening will be completed via a REDCap form or phone screening and will include measures of sociodemographic characteristics, the REALM-SF [31], the CUDIT-SF [28], and tobacco use history.

#### Baseline Assessments

Following completion of the screening survey, participants will complete the Baseline Part 1 enrollment call, download the Insight Mobile Health Platform app, and complete the baseline survey. Participants will be instructed to complete the full baseline survey via the smartphone app within approximately 1 day of receiving the baseline survey code, although up to 7 days will be allowed for completion before participants are considered ineligible. This survey will assess sociodemographic and background characteristics; financial stress using the Financial Chronic Stress Scale [36]; household food insecurity assessed using the US Household Food Security Survey Module [37]; self-rated health [47]; height and weight; religion and political orientation/stress [38,39]; tobacco use history; nicotine dependence assessed using the Heaviness of Smoking Index [40]; withdrawal symptoms assessed using the Brief Wisconsin Smoking Withdrawal Scale 2-B [41]; depressive symptoms assessed using the Center for Epidemiological Studies Depression Scale-10 (CES-D-10) [44]; alcohol use disorder via the Alcohol Use Disorders Identification Test-Consumption [45] and alcohol use and frequency using a 7-day Timeline Follow-Back approach, capturing daily quantity and frequency of consumption and heavy drinking episodes [46]; marijuana/cannabis use behavior adapted from a statewide study on medical and recreational cannabis use in Oklahoma [60]; motivation/importance/readiness to quit smoking [48]; and smoking self-efficacy via an adapted version of the 9-item Smoking Self-Efficacy/Temptation Short Form [49]. After completing the baseline survey, participants will complete a videoconferencing appointment with study staff (Baseline Part 2A), during which they will submit a CO breath sample to biochemically verify current smoking status. Participants will then complete a medical screening appointment with the study physician via videoconference (Baseline Part 2B). During this visit, participants will complete additional assessments, including the Patient Health Questionnaire-9 (PHQ-9) [42,43] and detailed tobacco use history, and will be evaluated for varenicline eligibility. Only participants who meet all eligibility criteria, including medical clearance for varenicline use, will proceed to randomization.

#### Daily Assessments

Participants will complete brief daily diary assessments via the study smartphone app beginning 1 week before their scheduled quit date and continuing through 12 weeks postquit (13 weeks total). Participants will be prompted to complete the daily diary each morning and will be compensated US $1 per day for completion (up to US $92). Daily diaries (≤20 items depending on skip patterns; ≈1‐2 min to complete) will include assessments of tobacco, alcohol, and cannabis use during the previous day (ie, yesterday), medication use (varenicline and nicotine lozenges [for those assigned]), withdrawal symptoms via the Brief Wisconsin Smoking Withdrawal Scale 2-B [41], sleep duration and quality (items adapted from the Pittsburgh Sleep Quality Index) [52], and pain intensity and interference (items adapted from the Patient-Reported Outcomes Measurement Information System questionnaire) [53,54].

#### Weekly Assessments

Thirteen weekly questionnaires will be administered at 7-day intervals during the 12-week postquit treatment period (beginning 1 week after the scheduled quit date). Weekly survey items will be embedded within the daily survey and presented on prespecified assessment days. Participants will be compensated US $10 for each completed weekly survey and breath sample (up to US $130).

##### Smoking Status and Substance Use

Self-reported smoking status will be assessed for the past 24 hours, 7 days, and 30 days, and since the scheduled quit date. Use of electronic cigarettes, other tobacco products, and cannabis will also be assessed for the past 7 days. Smoking abstinence will be biochemically verified via CO breath sample, with abstinence defined as ≤6 ppm. Alcohol use and frequency during the previous week will also be assessed.

##### Medication Adherence

An adapted version of the Adherence to Refills and Medications Scale (ARMS) [50,51] will be used to assess adherence to varenicline and nicotine lozenges. A 7-item version of the scale will be administered weekly during the first 12 weeks after the scheduled quit date. The survey items are rated from 1 (none of the time) to 4 (all of the time), with lower scores indicating better adherence. Adherence to varenicline and nicotine lozenges (for those assigned) will be assessed separately.

##### Symptoms and Side Effects

Participants will be asked to indicate which symptoms they have experienced over the past week from a list of 18 symptoms, including increased blood pressure, fast heart rate, dizziness, difficulty sleeping, unusual or vivid dreams, irritability, loss of appetite, indigestion/heartburn, hiccups, nausea, vomiting, constipation, gas, headache, fatigue, feeling sad, depression, worry, feeling anxious, or other (participant may specify). Participants will rate the severity of all selected symptoms as mild, moderate, or severe. Severe symptoms will trigger an encrypted email alert to study staff for follow-up. Participants may also indicate whether they would like to be contacted regarding any reported moderate symptoms at the conclusion of the measure. This assessment will provide information about potential medication side effects and/or may reflect nicotine withdrawal symptoms.

##### Motivation and Self-Efficacy

Self-efficacy for smoking abstinence will be assessed using an adapted version of the 9-item Smoking Self-Efficacy/Temptation Short Form [49], which measures participants’ confidence in their ability to cope with high-risk situations without relapsing. Motivation to quit smoking will be assessed using a 4-item Motivation/Importance/Readiness questionnaire (adapted from Crittenden et al [48]) to evaluate participants’ perceived importance of quitting, readiness to quit, and confidence in their ability to quit.

##### Mental Health and Substance Use

Mental health will be assessed using the PHQ-8 [42,43] depression screener, which excludes the suicidal ideation item, and the 10-item CES-D-10 [44], which assesses depressive symptoms over the past week.

##### Treatment Perceptions and Usability

Participants’ perceptions of counseling credibility will be assessed using a 3-item measure adapted from the Credibility/Expectancy Questionnaire [55]. The Credibility/Expectancy Questionnaire will be administered weekly from the quit day through 4 weeks postquit (when counseling concludes). Therapeutic alliance will be assessed using the Working Alliance Inventory-Short Revised [56,57], adapted to refer to “counseling” rather than “therapy” and “my counselor” rather than the counselor’s name. The Working Alliance Inventory-Short Revised will be administered at 1 and 4 weeks postquit. Perceptions of smartphone intervention usability will be assessed using the System Usability Scale [58,59], which was adapted to reference the study smartphone app.

##### Other Measures

Additional weekly measures will include self-rated health [47], weight (lb), and political stress (overall and in response to news media exposure).

##### Interim Assessments

Additional brief follow-up assessments will be administered at 17, 20, and 23 weeks postquit, with participants compensated US $5 per assessment (up to US $15). Measures will include self-reported smoking status (previous day, past 7 days, and since the scheduled quit date), 7-day tobacco/nicotine product use, and biochemically verified abstinence via CO breath sample (≤6 ppm). Alcohol use and frequency, cannabis use, depressive symptoms (CES-D-10 [44] and PHQ-8 [42,43]), and motivation/importance/readiness [48] will also be assessed.

### Final Follow-Up Assessment

The final follow-up assessment will be conducted at 26 weeks postquit. This assessment will include measures of tobacco and cannabis use, including the CUDIT-SF [28], and self-reported smoking status will be biochemically verified via a Bluetooth-enabled CO breath monitor. Participants will also complete measures of mental health and substance use, including the PHQ-8 [42,43], the CES-D-10 [44], the Alcohol Use Disorders Identification Test-Consumption [45], and alcohol quantity and frequency. Motivation, importance, and readiness to quit smoking [48] will be reassessed. Additional measures will include self-reported weight, self-rated health, the Financial Chronic Stress Scale [36], and the US Household Food Security Survey Module [37].

### Outcomes

#### Primary and Secondary Outcomes

The primary aim of this trial is to evaluate whether the combination of varenicline and oral NRT (VAR+NRT) increases the likelihood of achieving biochemically verified smoking cessation compared with varenicline alone. The primary outcome for the medication treatment factor (VAR+NRT vs VAR) will be CO-confirmed 7-day point prevalence abstinence at 26 weeks (consistent with current recommendations) [61]. Daily smoking behavior will also be monitored via smartphone-based assessments. Secondary analyses for Aim 1 will examine the effect of pharmacological treatment (VAR+NRT vs VAR) on earlier smoking cessation outcomes (eg, 7-day and 30-day point prevalence abstinence at the end of treatment) as well as daily reports of smoking abstinence during the 12-week treatment period.

Aim 2 will evaluate the effect of the smartphone medication adherence app (COMBO-A vs COMBO-B) on 2 primary outcomes: medication adherence during the 12-week treatment period and biochemically verified smoking cessation at 26 weeks postquit. Medication adherence will be measured weekly using an adapted version of the ARMS [50,51] and daily via smartphone-based assessments. Secondary analyses will evaluate the influence of the medication adherence app on earlier smoking cessation follow-up points and daily reports of smoking abstinence and medication adherence during the treatment period. Finally, Aim 3 will compare weekly symptom frequency and severity (including withdrawal symptoms and common medication side effects) between the treatment groups during the intervention period.

#### Analysis Plan

##### Factorial Analysis Approach

The 2 × 2 factorial design will be used to evaluate the main effects of the 2 randomized treatment factors: medication type (VAR+NRT vs VAR) and smartphone adherence intervention (COMBO-A vs COMBO-B). The study was powered separately to evaluate the main effect of each treatment factor on CO-confirmed 7-day point prevalence abstinence at 26 weeks, using a 2-sided significance level of .05 for each comparison, as described in Aims 1 and 2. The study was not specifically powered to detect an interaction between the 2 factors. The interaction between medication type and smartphone intervention will therefore be evaluated separately as an exploratory analysis.

##### Aim 1: Effect of VAR+NRT Versus VAR Alone on Smoking Cessation Outcomes

The power calculation (conducted using R software [version 4.0.5; R Foundation for Statistical Computing] and the *powerMediation* package [version 0.3.4; CRAN]) assumes a total sample size of 496 participants (248 per treatment factor). This estimate is based on expected 7-day biochemically verified point prevalence abstinence rates at the 26-week follow-up, with an anticipated 10% higher cessation rate in the VAR+NRT group compared with VAR alone (25% vs 15%). In a preliminary pilot study [14], participants assigned to VAR+oral NRT had a 20% cessation rate at 26 weeks, compared with 0% among those assigned to VAR alone. Given the small pilot sample, we conservatively estimate a 10% difference based on standard pharmacotherapy and counseling in the Tobacco Treatment Research Program, which typically results in a 15% abstinence rate. With these assumptions and a 2-sided significance level of .05, this sample size provides 80% power to detect a 10% difference in abstinence rates and would also provide adequate power to detect differences of 20% versus 10% or 20% versus 0%.

Logistic regression analyses will be conducted to evaluate the effect of VAR+NRT versus VAR alone on biochemically confirmed 7-day point prevalence smoking abstinence (binary) at 26 weeks postquit (primary outcome). Secondary outcomes will include prolonged abstinence, 30-day point prevalence abstinence at 26 weeks, and 7-day and 30-day point prevalence abstinence at 12 weeks postquit (end of treatment). Covariates may include age, sex, race/ethnicity, nicotine dependence, income, and education.

Generalized linear mixed models (GLMMs) will be used to evaluate the effect of treatment group (VAR+NRT vs VAR) on daily (self-reported) and weekly (biochemically verified 7-day point prevalence) smoking status as repeated outcome measures, while accounting for covariates and time since the scheduled quit date. GLMMs are a flexible analytic approach widely used in health sciences research [62]. GLMMs can handle fixed and random effect model parameters, nested designs, and repeated measures with various correlation structures [63,64]. GLMMs can also handle normal and nonnormal outcomes, including dichotomous and count-based smoking status variables, different variance functions, and unbalanced designs in which the number of repeated observations varies across individuals.

##### Missing Data and Dropouts

The primary analysis will follow an intention-to-treat approach, in which all randomized participants will be included in the group to which they were assigned. Participants who do not provide biochemical verification of abstinence at the 26-week follow-up will be classified as smoking for the primary analysis. However, because this approach has limitations, particularly when dropout rates differ between the interventions being compared [65], sensitivity analyses will be conducted using multiple imputation under a missing-at-random assumption, incorporating relevant smoking and demographic characteristics.

##### Aim 2: Effect of the Smartphone Medication Adherence App on Smoking Cessation and Medication Adherence

Logistic regression analyses will be conducted to evaluate the effect of COMBO-A versus COMBO-B on biochemically confirmed 7-day point prevalence smoking abstinence (binary) at 26 weeks postquit (primary outcome). As in Aim 1, secondary outcomes will include continuous/prolonged abstinence, 30-day point prevalence abstinence at 26 weeks, and 7-day point prevalence, continuous, and 30-day point prevalence abstinence at 12 weeks postquit date (end of treatment). Covariates may include age, sex, race/ethnicity, nicotine dependence, income, and education.

GLMMs will be used to evaluate the effect of the medication adherence app (COMBO-A vs COMBO-B) on repeated measures of smoking status and medication adherence. These measures will include weekly assessments of abstinence status (biochemically verified 7-day point prevalence), ARMS scores for varenicline and nicotine lozenges, and daily self-reports of smoking abstinence, varenicline adherence, and number of nicotine lozenges used. Measures related to nicotine lozenge use will be analyzed within the subsample assigned to VAR+NRT (n=248), whereas other outcomes will be analyzed in the full sample.

We will explore interactions between medication type (VAR vs VAR+NRT) and adherence intervention (COMBO-A vs COMBO-B) in predicting smoking cessation and medication adherence using regression models appropriate for each outcome (eg, logistic and mixed-effects models). These interaction analyses are exploratory and were not the basis of the study’s power calculations.

##### Aim 3: Compare Weekly Symptoms Between the Treatment Groups

GLMM analyses will be conducted to compare weekly symptom frequency and severity between treatment groups using data from the weekly symptom checklist, which assesses the presence and severity of 18 symptoms that may reflect medication side effects or withdrawal symptoms. Plausibly, participants assigned to VAR+NRT may experience lesser withdrawal symptom severity than those assigned to VAR, since they are able to address breakthrough symptoms with oral NRT as needed. Likewise, those assigned to COMBO-A may have lesser withdrawal symptom severity than COMBO-B because they may be more adherent to their medication(s). On the other hand, it is possible that those assigned to VAR+NRT might experience more weekly symptoms (ie, potential side effects) than those assigned to VAR alone. We will conduct additional exploratory analyses focused on evaluating individual group comparisons and medication adherence as a mediator of medication type and smoking cessation. Possible moderating effects of the covariates on the impact of each treatment factor (medication and smartphone intervention types) on cessation and medication adherence will also be explored.

Additionally, participants in the most intensive treatment (VAR+NRT+COMBO-A) will be compared with those in the least intensive treatment (VAR+COMBO-B) on smoking cessation and medication adherence outcomes. Medication adherence indicators (ie, proportion of days of complete adherence and weekly ARMS scores) will also be evaluated as potential mediators of the adherence intervention (COMBO-A vs COMBO-B) and smoking cessation outcomes [66].

### Ethical Considerations

This study protocol was approved by the institutional review board of the University of Oklahoma Health Campus (institutional review board number 17722). All procedures will be conducted in accordance with the ethical standards of the institutional research committee. All participants will provide informed consent prior to participation.

## Results

This study was funded by the National Cancer Institute in 2025. Recruitment began in July 2026, and as of this protocol submission, 8 participants have been enrolled. The trial aims to enroll 496 participants. Enrollment is expected to be completed by December 2028, with primary data collection and follow-up completed by June 2029. Primary study results are anticipated later in 2029.

## Discussion

Building on our pilot work [14], this randomized controlled trial will be the first to evaluate the efficacy of varenicline plus nicotine lozenges in a full-scale randomized trial. Likewise, this study will evaluate a smartphone app designed to improve tobacco cessation pharmacotherapy adherence. The study employs fully remote assessment and treatment delivery via telephone and a smartphone-based smoking cessation app, thus reducing barriers to treatment, expanding reach, and improving measurement validity. It is hypothesized that varenicline plus nicotine lozenges will lead to higher abstinence rates compared with varenicline alone at 26 weeks postquit. Additionally, participants receiving the smartphone intervention with adherence components (COMBO-A) are expected to demonstrate better medication adherence during the 13-week treatment period and higher cessation rates at 26 weeks postquit compared with those assigned to the basic smartphone intervention (COMBO-B).

Several key decisions were made in designing this trial. First, an efficient factorial study design is employed, which allows for the evaluation of 2 treatment interventions (pharmacological and smartphone medication adherence) in the same trial, thus reducing the number of participants who would be needed to evaluate these interventions in separate trials. The primary outcome, 7-day point prevalence abstinence, was selected because it is widely used in smoking cessation research [61] and accounts for delayed quit attempts and/or relapse followed by a return to abstinence. However, additional cessation measures, such as prolonged abstinence, 30-day point prevalence, and repeated assessments, will be evaluated as secondary and exploratory outcomes. While research suggests that nicotine lozenges and gum both have comparable effects on smoking cessation [67], lozenges offer some advantages. For example, lozenges are easier to use for individuals with dental problems and have better nicotine absorption compared with gum [13].

Combining varenicline with nicotine lozenges may improve cessation rates compared with varenicline alone. If found to be effective, adults who smoke will have an additional evidence-based treatment option to help them quit. Medication regimens requiring multiple daily doses can be challenging to follow, potentially reducing their effectiveness. Smartphone-based adherence interventions can provide a flexible and scalable solution to improve both medication adherence and smoking cessation outcomes. If effective, the smartphone adherence app could be widely implemented in tobacco cessation efforts and adapted for other medication regimens, potentially extending its impact beyond smoking cessation.

## Supplementary material

10.2196/108379 Checklist 1 SPIRIT checklist.

10.2196/108379 Peer Review Report 1 Peer review report by National Cancer Institute grant R01CA298165.

## Abbreviations

ARMS: Adherence to Refills and Medications Scale
CES-D-10: Center for Epidemiological Studies Depression Scale-10
CO: carbon monoxide
CUDIT-SF: Cannabis Use Disorder Identification Test-Short Form
GLMM: generalized linear mixed model
NRT: nicotine replacement therapy
PHQ: Patient Health Questionnaire
REALM-SF: Rapid Estimate of Adult Literacy in Medicine-Short Form
VAR: varenicline
