# Supplementary material for: A Randomized Factorial Trial of Varenicline, Nicotine Lozenges, and Smartphone Medication Adherence Reminders for Smoking Cessation: Protocol for the COMBO II Study
Source: JMIR Res Protoc. 2026 Nov 2;15:e108379. doi: 10.2196/108379 (PMC13727020; doi:10.2196/108379)
Supplement: Supplementary file 2 [file resprot-v15-e108379-s002.pdf]

SUMMARY STATEMENT

(Privileged Communication)

PROGRAM CONTACT:
MARIA Roditis
240-276-5518
maria.roditis@nih.gov

Release Date: **06/24/2024**
Revised Date:

Application Number: **1R01CA298165-01**

Principal Investigators (Listed Alphabetically):

AHLUWALIA, JASJIT SINGH
KENDZOR, DARLA ELIZABETH (Contact)

Applicant Organization: UNIVERSITY OF OKLAHOMA HLTH SCIENCES CTR

Review Group: **IPTA**
Interventions to Prevent and Treat Addictions Study Section

Meeting Date: **06/13/2024**
Council: **OCT 2024**
Requested Start: **12/01/2024**
Opportunity Number: **PAR-24-072**
PCC: **CMTC**

Project Title: **A Randomized Factorial Trial of Varenicline with Nicotine Lozenges and a Smartphone Medication Adherence Intervention to Promote Smoking Cessation**
SRG Action: **Impact Score:23 Percentile:8**
Next Steps: **Visit https://grants.nih.gov/grants/next_steps.htm**
Human Subjects: 30-Human subjects involved - Certified, no SRG concerns
Animal Subjects: 10-No live vertebrate animals involved for competing appl.
Gender: 1A-Both genders, scientifically acceptable
Minority: 1A-Minorities and non-minorities, scientifically acceptable
Age: 3A-No children included, scientifically acceptable

Project Year	Direct Costs Requested	Estimated Total Cost
1	499,308	730,239
2	499,544	730,584
3	499,544	730,584
4	499,544	730,584
5	499,926	731,142
TOTAL	2,497,866	3,653,132

ADMINISTRATIVE BUDGET NOTE: The budget shown is the requested budget and has not been adjusted to reflect any recommendations made by reviewers. If an award is planned, the costs will be calculated by Institute grants management staff based on the recommendations outlined below in the COMMITTEE BUDGET RECOMMENDATIONS section.

1R01CA298165-01 Kendzor, Darla

COMMITTEE BUDGET RECOMMENDATIONS

RESUME AND SUMMARY OF DISCUSSION: The goal of the proposed study is to evaluate the efficacy of combination varenicline plus nicotine lozenges relative to varenicline alone for smoking cessation, and the impact of a smartphone-based medication adherence intervention on medication adherence and smoking cessation. The project's high significance lies in its ability to advance what is currently known about addition of oral nicotine replacement therapy (NRT) to varenicline for improving smoking cessation rates and whether a mobile intervention can improve adherence to pharmacotherapy, with adherence being a significant barrier to successful smoking cessation for both varenicline and NRT. The investigative team was considered excellent, with the expertise and prior experience necessary to successfully complete this project, and with access to an excellent and well-resourced research environment. The project was considered highly innovative, being the first large-scale RCT comparing varenicline to varenicline+oral NRT, with the proposed novel way to address adherence, and with the fully remote protocol. The panel was enthusiastic about the many strengths of the application, including the strong scientific premise, the promising preliminary data, and the strong data analysis plan. During the discussion, the panel also noted some addressable weaknesses in the approach, including insufficient mechanism to validate subjects' adherence to medication, with self-reports being possibly impacted by social desirability. Concerns were also raised around the required heavy engagement from the app users. Following the discussion, the panel agreed that the weaknesses identified were minor and addressable and did not detract from the project's high impact. Overall, this well-articulated proposal was seen as potentially very important and with high probability of success. Findings are expected to have a high overall impact on the field.

DESCRIPTION (provided by applicant): Although the prevalence of smoking has declined among U.S. adults, smoking remains the leading preventable cause of cancer incidence and mortality. Quitting smoking increases life expectancy, and quitting at an earlier age is associated with more years of life gained. Effective pharmacotherapies to aid cessation are available, and the combination of behavioral support with pharmacotherapy optimizes cessation outcomes. Varenicline is an effective, first-line smoking cessation treatment, and recent research has investigated combination pharmacotherapy to improve the modest quit rates observed with monotherapy. The findings of two meta- analyses have indicated that varenicline combined with the nicotine patch was more effective than varenicline alone for smoking cessation However, no published studies to date have evaluated the combined impact of varenicline and oral nicotine replacement therapy (NRT) on smoking cessation in a randomized trial. Oral NRT, such as nicotine lozenges, can provide acute relief from cravings/withdrawal, and offers individuals the flexibility to deliver nicotine quickly, in contrast with the continuous, passive, and slow-acting delivery of the nicotine patch. Nevertheless, in clinical trials of other combination pharmacotherapies, participants' adherence to of oral NRT has been suboptimal, making it difficult to determine whether there is an added benefit. Given the near-ubiquity of smartphone ownership (85% of U.S. adults), it is plausible that smartphone-based medication adherence interventions could have a positive influence on pharmacotherapy adherence and smoking cessation. This investigative team has demonstrated the feasibility and potential efficacy of using combination varenicline plus oral NRT treatment to promote smoking cessation in a pilot factorial randomized trial. Likewise, pilot findings showed that medication adherence and smoking cessation rates were higher among those who received smartphone-based medication reminders than those who did not. The proposed study will enroll 496 adults who smoke cigarettes daily. The study will employ a 2x2 factorial design in which participants will be randomized to one of four combinations of two treatment factors: 1) pharmacological treatment (varenicline + nicotine lozenges vs. varenicline alone) and 2) smartphone medication adherence intervention (smartphone-based smoking cessation app with vs. without adherence

components). The primary study outcomes will be biochemically- confirmed 7-day point prevalence abstinence at 26 weeks after a scheduled quit date, and medication adherence over the 13-week treatment period. Smartphone-based daily diaries will be employed to assess daily smoking and medication adherence. Notably, the proposed study will employ entirely remote assessment and treatment delivery strategies. Exploratory analyses will evaluate the potential interaction between medication type and the smartphone adherence intervention, and compare the influences of pharmacological treatment type and the medication adherence intervention on weekly physical symptoms (e.g., withdrawal, medication side effects). The overarching goal of the proposed research is to improve smoking cessation treatment and decrease cancer risk.

PUBLIC HEALTH RELEVANCE: Although the prevalence of smoking has declined among U.S. adults, smoking remains the leading preventable cause of cancer incidence and mortality. The purpose of the proposed study is to evaluate: 1) the efficacy of combination varenicline plus nicotine lozenges relative to varenicline alone for smoking cessation, and 2) the impact of a smartphone-based medication adherence intervention on medication adherence and smoking cessation. Study findings may lead to new evidence-based treatment options that improve smoking cessation rates and reduce tobacco-related cancer risk.

CRITIQUE 1

Significance: 3
Investigator(s): 1
Innovation: 2
Approach: 2
Environment: 1

Overall Impact: This MPI application from Drs. Kendzor and Ahluwalia proposes to run the first fully powered clinical trial evaluating whether the addition of oral NRT to varenicline improves smoking cessation rates, and also whether a mobile intervention can improve adherence rates and subsequent smoking cessation; both manipulations will be tested in a factorial design. The scientific premise for the study is solid, as 1) meta-analyses have found that supplemental NRT can improve cessation rates and 2) adherence continues to be a significant barrier to successful smoking cessation for both varenicline and NRT. The overall design of the study has several strengths including a remote trial, biochemical verification, and an integrated app that serves as the primary interface for participants to study-related procedures. The team and environment are strong, leaving little doubt that the trial will be successfully carried out within the funding period. One weakness that decreases enthusiasm slightly is that the trial is not powered to detect the interaction effect of NRT x Adherence conditions, but rather the power analysis focuses soley on main effects. Even with an underpowered interaction effect, the application does have the potential to significantly impact the field and improve overall cessation rates.

1. Significance:

Strengths

- There is a strong rationale for using NRT lozenges to supplement varenicline, as the NRT should ameliorate withdrawal-related symptoms that are not addressed by varenicline. Oral NRT has the potential to be more beneficial than the patch as it can be administered ad lib throughout the day.

- Adherence is a significant barrier for both NRT and varenicline, so the use of an app that increases adherence could have significant effects on successful smoking cessation attempts.
- Combining two factors (NRT supplementation and Adherence reminders) could have powerful effects on smoking cessation outcomes which would advance the field.

Weaknesses

- The effect size for oral NRT+varenicline compared to varenicline alone is small in the COMBO pilot trial and the phone reminders seem to have a much greater effect on adherence and cessation. There is some concern that the interaction of the two factors is not being considered as a primary aim.

2. Investigator(s):

Strengths

- MPI Kendzor is Professor of Family and Preventive Medicine at the University of Oklahoma Health Sciences Center who has expertise in clinical trials for smoking cessation particularly among socioeconomically disadvantaged populations and who led the COMBO pilot study on which the proposed study is based.
- MPI Ahluwalia is Professor of Behavioral and Social Sciences and Internal Medicine at Brown University who has expertise in conducting smoking cessation trials, particularly for African American smokers.
- Dr. Businelle is the Peggy and Charles Stephenson Endowed Chair in Cancer and Professor of Family and Preventive Medicine. Dr. Businelle directs the mHealth Shared Resource center and was responsible for the development of the mobile app to be used in the proposed study.
- The other team members add expertise in biostatistics, medical oversight, and collection and analysis of EMA data.

Weaknesses

- None.

3. Innovation:

Strengths

- First large-scale randomized trial comparing varenicline to varenicline+oral NRT.
- Use of fully remote procedures increases reach of study and may improve engagement.
- No other studies have examined whether an app-based intervention can increase adherence to pharmacotherapy for smoking cessation.

Weaknesses

- None.

4. Approach:

Strengths

- The integrated mHealth environment is a strength, as all study related procedures are performed in the context of this app, which reduces need for participants to learn and keep track of multiple inputs.

- Use of recommended best practices for assessment of alternative tobacco products and primary cessation outcomes are both strengths.
- Plan for biochemical verification of smoking status has been successful in prior study, suggesting that investigators should have no problem implementing for the proposed trial.

Weaknesses

- Given the factorial design, it’s not clear why the authors powered the study to detect main effects but not the interaction effect.

5. Environment:

Strengths

- The investigators have access to multiple resources at the University of Oklahoma Health Sciences Center including a cancer center and the Tobacco Treatment Research Program that Dr. Kendzor leads, which should provide a large population of smokers interested in quitting.
- The mHealth resource is supported locally at UOHSC, which should facilitate the integration of this platform in the study.

Weaknesses

- None.

Study Timeline:

Strengths

- The study timeline budgets time for subject recruitment and follow-up and analyses/manuscript preparation. Given the team’s prior experience recruiting into remote trials, there are on concerns with the proposed timeline.

Weaknesses

- None.

Protections for Human Subjects:

Acceptable Risks and/or Adequate Protections

Data and Safety Monitoring Plan (Applicable for Clinical Trials Only):

Acceptable

Inclusion Plans:

- Sex/Gender: Distribution justified scientifically
- Race/Ethnicity: Distribution justified scientifically
- For NIH-Defined Phase III trials, Plans for valid design and analysis: Not applicable
- Inclusion/Exclusion Based on Age: Distribution justified scientifically

Vertebrate Animals:

Not Applicable (No Vertebrate Animals)

Biohazards:
Not Applicable (No Biohazards)

Applications from Foreign Organizations:
Not Applicable (No Foreign Organizations)

Select Agents:
Not Applicable (No Select Agents)

Resource Sharing Plans:
Not Applicable (No Relevant Resources)

Authentication of Key Biological and/or Chemical Resources:
Not Applicable (No Relevant Resources)

Budget and Period of Support:
Recommend as Requested

CRITIQUE 2

Significance: 2
Investigator(s): 1
Innovation: 2
Approach: 2
Environment: 1

Overall Impact: This project proposes a 2x2 factorial design study to study the efficacy of combined varenicline and oral NRT and smartphone medication reminders. The proposal presents results from a small-size (N=34) pilot study that used the same design; preliminary results are encouraging and form the basis of the power calculations. The investigative team is strong and has complementary expertise in tobacco cessation interventions, pharmacological trials, mobile health assessment/intervention, statistics, and prior collaborative work in the pilot study mentioned above. The power design for the study is clearly presented. The presented statistical methods are straightforward and appropriate for the study. The results of this study can improve smoking cessation strategies and ultimately decrease tobacco-related cancer risk.

1. Significance:

Strengths

- Unique study for investigating the efficacy of the combination therapy of varenicline and oral NRT.

- Smoking is still the leading cause of cancer incidence and mortality.
- Oral NRT could assist in managing acute craving episodes as a supplement to varenicline.
- Medication adherence app can be used widely to manage other tobacco cessation pharmacotherapy.

Weaknesses

- The mobile app and the mobile sensor component require robust engagement which might contribute to reduced retention.

2. Investigator(s):

Strengths

- The investigative team is strong and has complementary expertise in tobacco cessation interventions, pharmacological trials, mobile health assessment/intervention, and statistics, as well as prior collaborative work in the pilot study that forms the basis of the proposal.

Weaknesses

- None noted.

3. Innovation:

Strengths

- First large scale study designed to evaluate the efficacy of varenicline combined with oral NRT and smartphone app designed to improve tobacco cessation pharmacotherapy adherence.
- The study is conducted completely remotely.
- Built on mature smartphone mobile health platform.

Weaknesses

- None noted.

4. Approach:

Strengths

- Simple 2x2 factorial design to study the efficacy of combined varenicline and oral NRT and smartphone medication reminders.
- The proposal presents results from a small-size (N=34) pilot study that used the same design; preliminary results are encouraging and form the basis of the power calculations.
- Study conducted completely remotely that includes telephone-based smoking cessation counseling; mailed pharmacotherapy; and smartphone-based assessments and carbon monoxide breath sample submissions.
- The 2x2 factorial study is efficient as it allows for the evaluation of two treatment interventions in the same trial.

Weaknesses

- There is no mechanism to validate the adherence of the subjects to medication.

5. Environment:

Strengths

- Adequate resources are present. The app development is mature, prior collaboration experience.

Weaknesses

- None noted.

Study Timeline:

Strengths

- Clearly defined factorial study with achievable milestones and recruitment.

Weaknesses

- None noted.

Protections for Human Subjects:

Acceptable Risks and/or Adequate Protections

Data and Safety Monitoring Plan (Applicable for Clinical Trials Only):

Inclusion Plans:

- Sex/Gender: Distribution justified scientifically
- Race/Ethnicity: Distribution justified scientifically
- For NIH-Defined Phase III trials, Plans for valid design and analysis: Not applicable
- Inclusion/Exclusion Based on Age: Distribution justified scientifically
- Distribution is justified.

Vertebrate Animals:

Not Applicable (No Vertebrate Animals)

Biohazards:

Not Applicable (No Biohazards)

Applications from Foreign Organizations:

Not Applicable (No Foreign Organizations)

Select Agents:

Not Applicable (No Select Agents)

Resource Sharing Plans:

Acceptable

Authentication of Key Biological and/or Chemical Resources:
Not Applicable (No Relevant Resources)

Budget and Period of Support:
Recommend as Requested

CRITIQUE 3

Significance: 1
Investigator(s): 1
Innovation: 1
Approach: 4
Environment: 1

Overall Impact: The overall goal of this study is to assess whether combining varenicline with oral nicotine (vs. varenicline alone) and using medication adherence app (ADHERE vs. No ADHERE) will improve smoking cessation outcomes. The first aim is to evaluate if varenicline + oral nicotine is more effective than varenicline alone in smoking cessation assessed 26 weeks post quit date. The second aim is to compare the effects of ADHERE app vs. app without the medication adherence component on medication adherence during the treatment period and biochemically verified smoking cessation at 26 weeks after the quit date, and to explore the potential interaction between medication condition and medication adherence condition on these outcomes. The third aim is exploratory and will compare the influence of the medication and adherence interventions on weekly symptoms during the first 12 weeks after the quit date. This study will be conducted by an outstanding team of researchers who have the relevant expertise and experience to lead this study. They have already conducted a pilot study that provided strong preliminary data to support the full RCT. Additionally, the aim to enhance cigarette smoking cessation intervention using pharmacotherapy is highly significant. The combination of varenicline and oral nicotine as well as pairing it with medication adherence app is also a novel way to enhance smoking cessation interventions. Despite the high enthusiasm for this study, the overall impact is moderate due to issues with internal validity. There is no way to verify that participants are taking the medication other than relying on self-reports. There are no efforts to reduce social desirability bias. Such bias, if present, can lead to false interpretation of the results. There is also no consideration of whether counselors will be blinded to the participant condition and what the participants will be told about other tobacco use. Counselors knowing the participant’s randomized condition and use of other tobacco many compromise the study. If these concerns are addressed, this study has the potential to have high impact.

1. Significance:

Strengths

- Enhancing efforts to enhance quitting smoking using proven pharmacotherapies is important. Nicotine lozenges can help people deal with cravings and withdrawals, but the adherence has been low.
- Compelling case is provided for combining varenicline and NRT; varenicline and NRT promote smoking cessation via potentially different pathways and they may have different therapeutic

purposes. Varenicline may not fully saturate nicotine receptors so oral nicotine may provide relief from acute cravings.
- While NRT such as patches have been combined with varenicline, the combination of nicotine lozenges and varenicline has not been examined before.
- COM-B provides a strong theoretical basis for the medication adherence intervention.

Weaknesses
- None.

2. Investigator(s):

Strengths
- The research team is outstanding, composed of leaders in the field of smoking cessation and mobile health, with extensive experience recruiting smokers for RCTs.
- Dr. Kendzor is the founding director of the Tobacco Treatment Research Program (TTRP) which provides tobacco cessation treatment as well as conducts research. Dr. Ahluwalia has led tobacco cessation pharmacotherapy clinical trials. Both MPIs have worked together and have publications together.
- Co-Is have unique and necessary roles. Dr. Businelle developed the Insight mHealth platform that will be used in this study. Dr. Frank-Pearce is a biostatistician on this project and has worked with the study team in the past. Dr. Baranskaya will serve as a study physician at Oklahoma. Dr. Hebert has experience with using EMA data in research and will lead the analysis of EMA data.
- The team collaborated on the pilot study that serves as the basis for the current proposal.

Weaknesses
- Dr. Sifat’s unique role on the study is less clear. Their contribution seems to be the analysis of the COMBO study which was the basis for the current proposal.

3. Innovation:

Strengths
- The combination of nicotine lozenge and varenicline as well as the incorporation of a smart phone app to enhance medication adherence is unique.

Weaknesses
- None.

4. Approach:

Strengths
- Strong pilot data that serve as basis for this study showed that varenicline + oral NRT + and smartphone reminders produced greater cessation rates than varenicline alone.
- Sufficient justification for focusing on individuals who smoke and live in Oklahoma is provided. The team has extensive experience recruiting people who smoke living in OKL.
- Careful consideration of cannabis smoking and assessment of other tobacco use is provided.

- The mobile health platform is highly sophisticated and has features with different/relevant topics/content based on theoretical factors.

Weaknesses

- There are some important internal validity issues: 1) the medication adherence reminders texts are supposed to encourage people to use the medications. However, there is no way to objectively measure whether people are taking their medications. Medication adherence is measured by a questionnaire which could be easy to falsify. E.g., some may not want to fill out/get additional questions/messages so they may report that they are taking their medications or report adherence due to social desirability bias. There is a possibility that higher abstinence and adherence could be related to frequent contact and not the ADHERE intervention. 2) there is no mention of whether counselors will be blinded to the conditions. Counselors who work with participants throughout the treatment can inadvertently introduce bias.
- It's unclear what participants will be told about e-cigarettes and other tobacco product use during treatment. It is possible that participants may use other alternative tobacco products like e-cigarettes to quit during treatment period. Such use may interfere with how people use pharmacotherapy. Although e-cig and other tobacco use will be measured and assessed in the study, there is no instructions given to participants about other tobacco use nor will this be controlled for in the primary and secondary analyses.
- It appears that participants set a quit date once they start treatment but it's unclear when this is supposed to happen. This matters because the assessments revolve around the quit date.

5. Environment:

Strengths

- OUHSC provides excellent scientific environment to conduct this study.

Weaknesses

- None.

Study Timeline:

Strengths

- The study timeline is reasonable and appropriate to achieve the study aims.

Weaknesses

- None.

Protections for Human Subjects:

Acceptable Risks and/or Adequate Protections

- There are adequate protections for human participants.

Data and Safety Monitoring Plan (Applicable for Clinical Trials Only):

Acceptable

- o Data and safety monitoring plan is sufficient and the team will create a data and safety monitoring board.

Inclusion Plans:

- Sex/Gender: Distribution justified scientifically
- Race/Ethnicity: Distribution justified scientifically
- For NIH-Defined Phase III trials, Plans for valid design and analysis: Not applicable
- Inclusion/Exclusion Based on Age: Distribution justified scientifically
- Plans to recruit adults >18 years old and sex/gender & race are adequately justified.

Vertebrate Animals:
Not Applicable (No Vertebrate Animals)

Biohazards:
Not Applicable (No Biohazards)

Applications from Foreign Organizations:
Not Applicable (No Foreign Organizations)

Select Agents:
Not Applicable (No Select Agents)

Resource Sharing Plans:
Not Applicable (No Relevant Resources)

Authentication of Key Biological and/or Chemical Resources:
Not Applicable (No Relevant Resources)

Budget and Period of Support:
Budget Modifications Recommended (in amount/time)
Recommended budget modifications or possible overlap identified:

- 150 smart phones are requested. However, it's unclear if such large quantity is necessary considering that most individuals, including those in the low SES groups already own smartphones.

THE FOLLOWING SECTIONS WERE PREPARED BY THE SCIENTIFIC REVIEW OFFICER TO SUMMARIZE THE OUTCOME OF DISCUSSIONS OF THE REVIEW COMMITTEE, OR REVIEWERS' WRITTEN CRITIQUES, ON THE FOLLOWING ISSUES:

PROTECTION OF HUMAN SUBJECTS: ACCEPTABLE

INCLUSION OF WOMEN PLAN: ACCEPTABLE

INCLUSION OF MINORITIES PLAN: ACCEPTABLE

INCLUSION ACROSS THE LIFESPAN: ACCEPTABLE

COMMITTEE BUDGET RECOMMENDATIONS: See comments from Rev. 3.

Footnotes for 1R01CA298165-01; PI Name: Kendzor, Darla Elizabeth

NIH has modified its policy regarding the receipt of resubmissions (amended applications).See Guide Notice NOT-OD-18-197 at https://grants.nih.gov/grants/guide/notice-files/NOT-OD-18-197.html. The impact/priority score is calculated after discussion of an application by averaging the overall scores (1-9) given by all voting reviewers on the committee and multiplying by 10. The criterion scores are submitted prior to the meeting by the individual reviewers assigned to an application, and are not discussed specifically at the review meeting or calculated into the overall impact score. Some applications also receive a percentile ranking. For details on the review process, see http://grants.nih.gov/grants/peer_review_process.htm#scoring.

MEETING ROSTER

Interventions to Prevent and Treat Addictions Study Section
Risk, Prevention and Health Behavior Integrated Review Group
CENTER FOR SCIENTIFIC REVIEW
IPTA
06/13/2024 - 06/14/2024

Notice of NIH Policy to All Applicants: Meeting rosters are provided for information purposes only. Applicant investigators and institutional officials must not communicate directly with study section members about an application before or after the review. Failure to observe this policy will create a serious breach of integrity in the peer review process, and may lead to actions outlined in NOT-OD-22-044 at https://grants.nih.gov/grants/guide/notice-files/NOT-OD-22-044.html, including removal of the application from immediate review.

CHAIRPERSON(S)

PIPER, MEGAN E, PHD
PROFESSOR
DEPARTMENT OF MEDICINE
UNIVERSITY OF WISCONSIN-MADISON
MADISON, WI 53711

MEMBERS

AUDRAIN-MCGOVERN, JANET, PHD
PROFESSOR AND DIRECTOR
DEPARTMENT OF PSYCHIATRY
PERELMAN SCHOOL OF MEDICINE
UNIVERSITY OF PENNSYLVANIA
PHILADELPHIA, PA 19104

BINSWANGER, INGRID A, MD
SENIOR CLINICIAN INVESTIGATOR
INSTITUTE FOR HEALTH RESEARCH
KAISER PERMANENTE
AURORA, CO 80014

BREWER, JUDSON A, PHD, MD *
PROFESSOR
DEPARTMENT OF BEHAVIORAL AND SOCIAL SCIENCES
DEPARTMENT OF PSYCHIATRY
BROWN UNIVERSITY
PROVIDENCE, RI 02903

CLAUS, ERIC D, PHD
ASSOCIATE PROFESSOR
DEPARTMENT OF BIOBEHAVIORAL HEALTH
THE PENNSYLVANIA STATE UNIVERSITY
UNIVERSITY PARK, PA 16802

COCHRAN, GERALD T, PHD *
PROFESSOR
DEPARTMENT OF INTERNAL MEDICINE
DIVISION OF EPIDEMIOLOGY
UNIVERSITY OF UTAH
SALT LAKE CITY, UT 84132

DIBELLO, ANGELO MICHAEL, PHD *
ASSISTANT PROFESSOR
DEPARTMENT OF GRADUATE SCHOOL OF APPLIED AND PROFESSIONAL PSYCHOLOGY
RUTGERS UNIVERSITY
PISCATAWAY, NJ 08854

EBRAHIMI KALAN, MOHAMMAD, PHD *
ASSISTANT PROFESSOR
SCHOOL OF HEALTH PROFESSION
EASTERN VIRGINIA MEDICAL SCHOOL,
NORFOLK, VA 23507

ERTIN, EMRE, PHD *
ASSOCIATE PROFESSOR
DEPARTMENT OF ELECTRICAL AND COMPUTER ENGINEERING
COLLEGE OF ENGINEERING
THE OHIO STATE UNIVERSITY
COLUMBUS, OH 43210

FRIDBERG, DANIEL J, PHD *
ASSOCIATE PROFESSOR
DEPARTMENT OF PSYCHIATRY AND BEHAVIORAL NEUROSCIENCE
THE UNIVERSITY OF CHICAGO
CHICAGO, IL 60637

HAGLE, HOLLY, PHD *
ASSOCIATE RESEARCH PROFESSOR
MISSOURI CENTER FOR ADDICTION RESEARCH AND ENGAGEMENT
COLLEGE OF ARTS AND SCIENCE
UNIVERSITY OF MISSOURI
KANSAS CITY, MO 64110

KLEMPERER, ELIAS MUSHABAC, PHD *
ASSISTANT PROFESSOR
DEPARTMENT OF PSYCHIATRY
LARNER COLLEGE OF MEDICINE
UNIVERSITY OF VERMONT
BURLINGTON, VT 05405

KMIEC, JULIE, DO *
ASSOCIATE PROFESSOR
DEPARTMENT OF PSYCHIATRY
UNIVERSITY OF PITTSBURGH
PITTSBURGH, PA 15213

KONG, GRACE, PHD
ASSOCIATE PROFESSOR
DEPARTMENT OF PSYCHIATRY
YALE UNIVERSITY SCHOOL OF MEDICINE
NEW HAVEN, CT 06519

KOWALCHUK, ALICIA ANN, DO *
ASSOCIATE PROFESSOR
BAYLOR COLLEGE OF MEDICINE
HOUSTON, TX 77030

LEDGERWOOD, DAVID M, PHD
PROFESSOR
DEPARTMENT OF PSYCHIATRY
AND BEHAVIORAL NEUROSCIENCES
SCHOOL OF MEDICINE
WAYNE STATE UNIVERSITY
DETROIT, MI 48201

LEE, DUSTIN CLARK, PHD *
ASSISTANT PROFESSOR
DEPARTMENT OF PSYCHIATRY AND BEHAVIORAL
SCIENCES
THE JOHNS HOPKINS SCHOOL OF MEDICINE
BALTIMORE, MD 21224

LOPEZ-CASTRO, TERESA, PHD *
ASSOCIATE PROFESSOR
DEPARTMENT OF PSYCHOLOGY
CITY COLLEGE OF NEW YORK
NEW YORK, NY 10031

MARK, KATRINA, MD *
ASSOCIATE PROFESSOR
DEPARTMENT OF OBSTETRICS, GYNECOLOGY
AND REPRODUCTIVE SCIENCES
UNIVERSITY OF MARYLAND SCHOOL OF MEDICINE
BALTIMORE, MD 21201

MCHUGH, REBECCA KATHRYN, PHD
ASSOCIATE PROFESSOR
DEPARTMENT OF PSYCHIATRY
HARVARD MEDICAL SCHOOL
BELMONT, MA 02478

MCNEELY, JENNIFER, MD
ASSOCIATE PROFESSOR
DEPARTMENTS OF POPULATION HEALTH AND MEDICINE
GROSSMAN SCHOOL OF MEDICINE
NEW YORK UNIVERSITY
NEW YORK, NY 10016

MCPHERSON, STERLING M, PHD
DIRECTOR AND PROFESSOR
PROGRAM OF EXCELLENCE IN ADDICTIONS RESEARCH
DEPARTMENT OF COMMUNITY AND BEHAVIORAL HEALTH
ELSON S. FLOYD COLLEGE OF MEDICINE
WASHINGTON STATE UNIVERSITY
SPOKANE, WA 99210

MONTGOMERY, LATRICE, PHD
DIRECTOR OF CLINICAL RESEARCH AT RIA HEALTH,
ADJUNCT ASSOCIATE PROFESSOR
DEPARTMENT OF PSYCHIATRY AND
BEHAVIORAL NEUROSCIENCE
COLLEGE OF MEDICINE
UNIVERSITY OF CINCINNATI
CINCINNATI, OH 45229

OSTACHER, MICHAEL JOSHUA, MD, MPH *
PROFESSOR
DEPARTMENT OF PSYCHIATRY AND BEHAVIORAL
SCIENCES
STANFORD UNIVERSITY
PALO ALTO, CA 94304

RIDENOUR, TY A, PHD *
RESEARCH PUBLIC HEALTH ANALYST
CENTER FOR SOCIAL DETERMINANTS,
RISK FACTORS, AND PREVENTION SCIENCE
RESEARCH TRIANGLE INSTITUTE
RESEARCH TRIANGLE PARK, NC 27709

SADASIVAM, RAJANI, PHD
PROFESSOR
DIVISION OF HEALTH AND IMPLEMENTATION SCIENCE
DEPARTMENT OF POPULATION AND QUANTITATIVE
HEALTH SCIENCES
UNIVERSITY OF MASSACHUSETTS MEDICAL SCHOOL
WORCESTER, MA 01605

SCHWEBEL, FRANK, PHD *
RESEARCH ASSISTANT PROFESSOR
CENTER ON ALCOHOL, SUBSTANCE USE, AND ADDICTIONS
THE UNIVERSITY OF NEW MEXICO
ALBUQUERQUE, NM 87123

SCOTT, KELLI, PHD *
ASSISTANT PROFESSOR
DEPARTMENT OF MEDICAL SOCIAL SCIENCES
FEINBERG SCHOOL OF MEDICINE
NORTHWESTERN UNIVERSITY
CHICAGO, IL 60613

SPEARS, CLAIRE ADAMS, PHD
ASSOCIATE PROFESSOR
DEPARTMENT OF HEALTH POLICY & BEHAVIORAL SCIENCES
SCHOOL OF PUBLIC HEALTH
GEORGIA STATE UNIVERSITY
ATLANTA, GA 30303

SPILLANE, NICHEA SOLOMON, PHD
ASSOCIATE PROFESSOR
DEPARTMENT OF PSYCHOLOGY
COLLEGE OF HEALTH SCIENCES
UNIVERSITY OF RHODE ISLAND
KINGSTON, RI 02881

STEVENS-WATKINS, DANELLE J, PHD
PROFESSOR
DEPARTMENT OF EDUCATIONAL, SCHOOL, AND COUNSELING PSYCHOLOGY
COLLEGE OF EDUCATION
UNIVERSITY OF KENTUCKY
LEXINGTON, KY 40506

STORMSHAK, ELIZABETH A, PHD
PROFESSOR
COLLEGE OF EDUCATION
PREVENTION SCIENCE INSTITUTE
UNIVERSITY OF OREGON
EUGENE, OR 97403

WADE, NATASHA E, PHD *
ASSISTANT PROFESSOR
DEPARTMENT OF PSYCHIATRY
UNIVERSITY OF CALIFORNIA, SAN DIEGO
LA JOLLA, CA 92093

WASHINGTON, PETER, PHD *
ASSISTANT PROFESSOR
INFORMATION & COMPUTER SCIENCES DEPARTMENT
UNIVERSITY OF HAWAII AT MĀNOA
HONOLULU, HI 96822

WELSH, JUSTINE WITTENAUER, MD *
ASSOCIATE PROFESSOR
DEPARTMENT OF PSYCHIATRY AND BEHAVIORAL SCIENCES
EMORY UNIVERSITY SCHOOL OF MEDICINE
ATLANTA, GA 30307

YI, RICHARD, PHD
PROFESSOR AND DIRECTOR
COFRIN LOGAN CENTER FOR ADDICTION RESEARCH AND TREATMENT
DEPARTMENT OF PSYCHOLOGY
UNIVERSITY OF KANSAS
LAWRENCE, KS 66045

SCIENTIFIC REVIEW OFFICER

ZANDBERG, IZABELLA, PHD
SCIENTIFIC REVIEW OFFICER
CENTER FOR SCIENTIFIC REVIEW
BETHESDA, MD 20892

EXTRAMURAL SUPPORT ASSISTANT

AMARE, MERON ERMIAS
LEAD EXTRAMURAL SUPPORT ASSISTANT
CENTER FOR SCIENTIFIC REVIEW
NATIONAL INSTITUTES OF HEALTH
BETHESDA, MD 20892

* Temporary Member. For grant applications, temporary members may participate in the entire meeting or may review only selected applications as needed.

Consultants are required to absent themselves from the room during the review of any application if their presence would constitute or appear to constitute a conflict of interest.
